# Membrane-Targeted Consequences of Acetaminophen Toxicity and Off-Target Effects of Antimicrobial Peptides on Host Cell Membranes

**DOI:** 10.3390/ijms27146234

**Published:** 2026-07-13

**Authors:** Oksana M. Voloshchuk, Volodymyr Berest, Oleksii Skorokhod

**Affiliations:** 1 Department of Biochemistry and Biotechnology, Yuriy Fedkovych Chernivtsi National University, 2 Kotsiubynskoho St., 58012 Chernivtsi, Ukraine; o.voloschuk@chnu.edu.ua; 2 Department of Molecular and Medical Biophysics, School of Radio Physics, Biomedical Electronics and Computer Systems, V. N. Karazin Kharkiv National University, 4 Svobody Sq, 61022 Kharkiv, Ukraine; berest@karazin.ua; 3 Department of Life Sciences and Systems Biology, University of Turin, via Accademia Albertina 13, 10123 Turin, Italy

**Keywords:** acetaminophen toxicity, antimicrobial peptides AMPs, membrane damage, mitochondrial dysfunction, off-target effects, combined exposure

## Abstract

Acetaminophen (paracetamol, APAP) is a widely used analgesic and antipyretic drug. Under normal physiological conditions, it does not directly interact with or disrupt cellular membranes. However, in cases of acetaminophen overdose or toxicity, severe cellular damage has been described, involving a broad spectrum of effects at different cellular levels. These toxic effects may involve membrane structures, including mitochondrial, plasma, and other intracellular membranes. Antimicrobial peptides (AMPs) are short, usually cationic and amphipathic peptides produced by both microorganisms and multicellular organisms, serving diverse defensive and competitive functions. In many cases, they exert their antimicrobial activity by direct interaction with bacterial or fungal membranes, leading to membrane destabilization and cell death. Owing to this membrane-targeting mechanism, AMPs may also interact with eukaryotic cell membranes, thereby exerting toxic or off-target effects under certain conditions. Here, we review the current knowledge on the membrane-related effects of acetaminophen toxicity and the mechanisms by which AMPs interact with biological membranes. In the event of combined exposure to acetaminophen and AMPs in therapeutic or experimental settings, the biological consequences remain unexplored. Such combined exposure may give rise to toxic effects and membrane-associated alterations. We further discuss potential mechanisms of interference, additive toxicity, and synergistic interactions between acetaminophen and AMPs, highlighting critical knowledge gaps and directions for future research.

## 1. Introduction

Acetaminophen (N-acetyl-p-aminophenol, paracetamol, APAP) is a widely used analgesic and antipyretic drug. Its consumption is extensive worldwide, and in some settings patterns of habitual or excessive use have been reported, particularly due to its over-the-counter availability and frequent administration for pain and fever relief. Under normal physiological conditions and at therapeutic doses, APAP does not directly interact with or disrupt cellular membranes. However, in cases of acetaminophen overdose or toxicity, severe cellular damage has been described, involving a broad spectrum of effects at multiple cellular levels [1,2,3]. These toxic effects include alterations of membrane structures, particularly mitochondrial, plasma, and other intracellular membranes [4,5].

Synergistic drug interactions between APAP and several compounds have been partially characterized, including combinations of APAP with nonsteroidal anti-inflammatory drugs (NSAIDs), which produce pharmacological (multimodal analgesic) synergy [6,7], as well as metabolic interactions associated with enzyme induction and hepatotoxicity [8]. However, interactions between APAP and antimicrobial peptides (AMPs) or other membrane-active antibiotics have not yet been investigated.

Antibiotic therapy remains a critical pillar of modern medicine. Although prescribing practices have become more regulated in recent years, inappropriate and excessive antibiotic use remains a significant global issue, with estimates suggesting that up to 30% of antibiotics are used inappropriately, contributing to antimicrobial resistance and major public health concerns [9,10,11]. Among antimicrobial agents, AMPs represent a distinct class of antibiotics. AMPs are short, typically cationic and amphipathic peptides produced by microorganisms as well as multicellular organisms, where they serve diverse defensive and competitive functions. In many cases, they exert antimicrobial activity through direct interaction with bacterial or fungal membranes, leading to membrane destabilization and cell death. Due to this membrane-targeting mechanism, AMPs may also interact with eukaryotic cell membranes, potentially causing toxic or off-target effects under certain conditions. While interactions among AMPs are well documented, interactions between AMPs and other drugs remain far less explored. In particular, potential additive or synergistic effects between AMPs and various membrane-active or bioactive compounds may be of considerable scientific and therapeutic interest, although the underlying mechanisms remain largely unstudied.

Although a slight antimicrobial activity of APAP has been reviewed [11], the drug is more commonly co-administered with conventional antibiotics in clinical practice. Nevertheless, the biological consequences of combined exposure to acetaminophen and AMPs in therapeutic or experimental settings remain unexplored. Such co-exposure may give rise to toxic effects and membrane-associated alterations. However, studies specifically addressing potential interference, additive, or synergistic interactions between acetaminophen and AMPs are currently lacking and warrant further investigation.

Although membrane alterations are not considered the primary initiating events in APAP-induced hepatotoxicity, they represent important downstream processes that can substantially influence the progression and outcome of liver injury. Membrane alterations affect multiple cellular functions, including membrane integrity, transporter activity, cell signaling, inflammatory responses, and intercellular communication, and may also contribute to drug–drug interactions and therapeutic responses. Despite their potential biological and clinical significance, these membrane-associated processes have received considerably less attention than the well-established mechanisms of metabolic activation, mitochondrial dysfunction, and oxidative stress.

Accordingly, this review aims to explore the potential interactions between APAP and AMPs, with particular emphasis on their effects on cellular membranes. We discuss how membrane-related mechanisms may influence APAP-induced hepatotoxicity and evaluate whether combined exposure to APAP and AMPs could result in additive, synergistic, or otherwise modified toxic effects. By focusing on these understudied aspects, this review seeks to identify current knowledge gaps and highlight priorities for future research.

## 2. High-Dose APAP Toxicity

APAP continues to be the most prevalent pharmacological agent in global clinical practice, owing to its established analgesic and antipyretic efficacy [12]. As a non-opioid analgesic, it is indicated for the management of mild-to-moderate pain, including cephalalgia, postpartum distress, and myalgia, serving as a primary alternative when salicylate therapy is contraindicated or ineffective [13,14]. However, the therapeutic window is often compromised by patient non-compliance with dosing protocols, inadvertent polypharmacy involving multiple APAP-containing formulations, or chronic supratherapeutic ingestion [15]. Consequently, despite its high safety profile at standard doses, this compound represents the leading etiology of acute liver failure in Western nations and accounts for nearly half of all drug-induced liver injury (DILI) cases in the United States and parts of Europe, which significantly constrains its clinical application [8]. Although pediatric populations frequently experience paracetamol exposure, toxicological outcomes in adults are typically more severe and possess a higher mortality rate. APAP-induced toxicity may manifest following a single massive ingestion or as a result of cumulative high-dose administration. Observational evidence indicates that the prevalence of paracetamol-associated hepatotoxicity has increased markedly over recent decades, establishing it as the leading cause of acute liver failure in the United States, the United Kingdom, and several European countries [16]. Under conditions of acetaminophen-induced toxicity, leukocyte infiltration is often exacerbated, triggering the activation of monocytes and Kupffer cells. These cells subsequently discharge a surge of pro-inflammatory cytokines and chemokines, potentially culminating in a ‘cytokine storm’ that intensifies the systemic inflammatory response [17]. In severe cases, liver transplantation remains the only life-saving therapeutic option, underscoring the fact that APAP-induced liver injury continues to represent a major public health challenge [18].

Although antibiotics are not part of the standard treatment for APAP toxicity, antimicrobial therapy may become necessary in severe cases complicated by acute liver failure and secondary infections [19,20]. However, the potential synergistic effects and biological activities arising from interactions between APAP and antimicrobial agents remain poorly investigated.

APAP-associated hepatotoxicity and nephrotoxicity, together with the pathophysiological cascades leading to hepatocyte and renal cell injury and death, constitute a complex multistage process that is not yet fully understood. This process is characterized by APAP metabolic bioactivation into reactive intermediates, depletion of endogenous antioxidant defenses, severe mitochondrial dysfunction, and disruption of cellular membrane integrity (these mechanisms are discussed in detail in this review). Importantly, additional factors, including antibiotics in general and AMPs in particular, could potentially influence these pathological processes. Moreover, nutritional status and particularly protein malnutrition has been shown to modulate APAP pharmacokinetics and pharmacodynamics, further complicating these interactions and warranting additional investigation [21].

### 2.1. Molecular Architecture of Acetaminophen Metabolism and the Formation of the Toxic Threshold

APAP is one of the most widely used analgesic and antipyretic agents for the treatment of acute and chronic pain and fever. Although it is extensively used in clinical practice, acetaminophen exhibits complex metabolism, and the precise mechanisms underlying its analgesic and antipyretic effects remain incompletely understood [22,23]. APAP appears to exert its analgesic effects through at least two major mechanisms: modulation of cyclooxygenase (COX) activity and the actions of its bioactive metabolite N-arachidonoylphenolamine (AM404), which interacts with transient receptor potential vanilloid 1 (TRPV1) channels and cannabinoid receptors, and potentially other targets, within the brain and spinal dorsal horn [16,23].

Following oral administration, APAP is primarily absorbed via passive diffusion across the intestinal epithelium [21], reaching peak therapeutic plasma concentrations within 30–120 min. In overdose scenarios, peak plasma or blood concentrations are typically observed approximately 4 h after ingestion. The elimination half-life of acetaminophen is approximately 2 h under normal physiological conditions but may be prolonged to up to 17 h in patients with pre-existing hepatic dysfunction. In adults, the recommended therapeutic dose is 325–1000 mg every 4–6 h, with a maximum daily dose not exceeding 4 g to reduce the risk of hepatotoxicity [13,22]. In pediatric patients, dosing is weight-based, typically 10–15 mg/kg per dose administered every 4–6 h, with a maximum daily dose of approximately 75 mg/kg [22,24]. Increasingly, clinical guidelines and regulatory agencies advocate for a reduced maximum daily adult dose of 3 g, particularly in individuals with risk factors such as chronic alcohol consumption, malnutrition, or underlying liver disease, to enhance safety margins and minimize the incidence of acetaminophen-induced liver injury [14,25]. Ingestion exceeding 7.5–10 g within a 24 h period significantly escalates the risk of induced hepatotoxicity. Furthermore, the risk threshold lowers for patients consuming more than 4 g daily who possess susceptibility factors such as malnutrition, chronic ethanol consumption, fasting, or the use of cytochrome P450 (CYP)-inducing agents [14,26].

**Figure 1:**
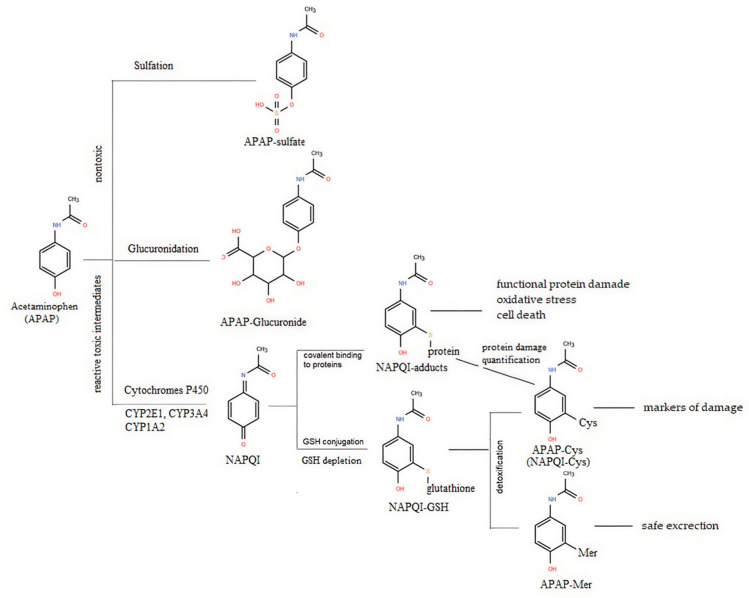
Metabolic pathways of acetaminophen. Major pathways involved in acetaminophen metabolism are illustrated. Key molecular and cellular reactions are indicated adjacent to the relevant intermediates and are discussed in detail in the main text. APAP: acetaminophen (paracetamol); NAPQI: N-acetyl-p-benzoquinone imine; GSH: glutathione; NAPQI-Mer: NAPQI-mercapturate; NAPQI-Cys: NAPQI-cysteine.

In the liver, acetaminophen undergoes extensive biotransformation designed to produce water-soluble, inert compounds (Figure 1). This multi-pathway process is primarily localized within the centrilobular region of the liver. The vast majority of the drug (85% to 90%) is processed through conjugation with glucuronic acid and sulfate [15] (Figure 1). Glucuronidation is mediated by the UDP-glucuronosyltransferase (UGT) family, particularly the UGT1A1, 1A6, and 1A9 isoforms, whereas sulfation is facilitated by sulfotransferases (SULT1A1, 1A3, and 2A1). These reactions occur mainly in the endoplasmic reticulum and cytosol, yielding metabolites excreted via the kidneys and bile through transport proteins such as MRP2 and MRP3. The sulfation biotransformation pathway is the first to achieve saturation during acetaminophen overdose, a phenomenon primarily driven by the finite supply of the required PAPS cofactor.

Glucuronide conjugates are rapidly exported from hepatocytes and are primarily excreted into the bile [27]. Most APAP sulfate conjugates are transported into the biliary system via multidrug resistance–associated protein 2 (MRP2), with a smaller fraction mediated by breast cancer resistance protein (BCRP/ABCG2) [28]. Only approximately 2% of acetaminophen is eliminated unchanged in the urine. The toxicological potential of acetaminophen arises from a minor oxidative metabolic pathway (approximately 5–10%) mediated by cytochrome P450 CYP enzymes, which generates reactive intermediates responsible for hepatocellular injury [29]. The CYP2E1 isoform plays a dominant role, although CYP1A2, CYP3A4, and CYP2D6 also contribute significantly [30,31]. Two-electron oxidation of acetaminophen generates a highly electrophilic intermediate: N-acetyl-p-benzoquinone imine (NAPQI), the primary driver of damage to the liver, kidneys, and other organs [31].

NAPQI is exceptionally reactive toward nucleophilic centers in cellular macromolecules, particularly the sulfhydryl (-SH) groups of cysteine. Under normal physiological conditions and therapeutic doses, NAPQI is instantly neutralized by conjugation with reduced glutathione (GSH), a pivotal tripeptide in antioxidant defense. This reaction can occur spontaneously or via glutathione-S-transferases (GST), resulting in the formation of non-toxic mercapturates excreted by the kidneys or bile [15]. A critical transition to toxicity occurs when NAPQI production outpaces the rate of glutathione synthesis and regeneration, often seen in overdose or during GSH depletion. Glutathione reserves are sensitive to nutritional status, with malnutrition leading to significant exhaustion [32]. When hepatic glutathione levels fall below approximately 30% of baseline, detoxification of the reactive NAPQI becomes insufficient. Accumulated NAPQI then covalently binds to protein thiol groups, forming acetaminophen–protein adducts that contribute significantly to hepatotoxicity [16]. This covalent interaction with bioactive molecules plays a central role in the pathogenesis of acetaminophen-induced injury. Both APAP and NAPQI are well known for their ability to form covalent adducts with cellular proteins [33,34]. In time-course study, the peak of protein covalent adduction typically precedes overt hepatic necrosis by 1–2 h; moreover, patients with the most severe toxicity exhibit the highest plasma levels of APAP–protein adducts, which correlate with elevations in serum transaminases [16]. Currently, APAP is known to form adducts with more than 30 hepatic proteins, including glutathione peroxidase (GPx), although the resulting inhibition of this enzyme appears to be moderate [1]. The activity of Na^+^/K^+^-ATPase and other ion pumps has also been reported to decrease following hepatotoxic APAP exposure, a phenomenon proposed to result from protein adduction, although direct evidence of covalent modification of these targets remains lacking [35]. Here, at the membrane level, APAP-induced alterations may be particularly relevant for potential interactions with AMPs. In addition, possible direct binding between APAP and AMPs should be investigated. Returning to the mechanisms underlying APAP toxicity, although covalent protein binding is considered a critical initiating event, it is widely accepted that this mechanism alone cannot fully explain the complexity of APAP-induced hepatotoxicity, which is recognized as a multifactorial process [3].

Measurement of APAP–protein adducts in plasma is currently considered the gold standard for confirming drug-induced liver injury (DILI) [34]. Tandem mass spectrometry (liquid chromatography–tandem mass spectrometry, LC–MS/MS) enables the detection of N-acetyl-p-benzoquinone imine (NAPQI) adducts bound to serum albumin, even after acetaminophen is no longer detectable in circulation [33]. While N-acetylcysteine (NAC) remains the standard antidote, its efficacy is constrained by an 8–16 h window. Recent research identifies fomepizole as a promising adjuvant, acting as a dual inhibitor that blocks CYP2E1 and suppresses the JNK signaling pathway, thereby preventing late-stage mitochondrial collapse [3].

Table 1 illustrates the distribution of acetaminophen metabolic fluxes as a function of dosage and the functional status of detoxification systems.

### 2.2. Mitochondrial Membrane Impairment as a Pivotal Link in Hepatotoxicity

The prevailing biochemical paradigm identifies mitochondria as the primary target during APAP-mediated hepatocyte death. Although NAPQI interacts with various cytosolic proteins, the formation of mitochondrial protein adducts correlates most closely with necrotic progression [3,36]. Consistent with this view, several authors have localized NAPQI-induced production of reactive oxygen species (ROS) and lipid peroxidation (LPO) to mitochondria, and have shown that scavenging mitochondrial ROS, peroxynitrite, and LPO products effectively prevents hepatocyte death [3,37,38,39]. Nitrotyrosine protein adducts were also detected within mitochondria. Additional evidence for selective APAP-induced mitochondrial oxidative stress includes the specific elevation of mitochondrial GSSG levels [38]. However, different authors differ in their evaluation of the relative contribution of ROS and LPO to cell injury following APAP overdose.

NAPQI selectively targets proteins in the mitochondrial matrix and inner membrane, including components of the electron transport chain (ETC) like complexes I and II, and the α-subunit of ATP synthase. The enzyme 3-hydroxy-3-methylglutaryl-CoA synthase 2 is particularly vulnerable, with its activity dropping sharply due to thiol modification [39]. Recent proteomic research has identified other NAPQI-modified proteins, such as peroxiredoxin 6, the redox-sensitive chaperone PARK7, and the voltage-dependent anion channel VDAC2 [40]. NAPQI binding to mitochondrial proteins demonstrates a significant correlation with the severity of APAP toxicity [3]. The detection of APAP adducts on ATP synthase supports the hypothesis that the initial source of mitochondrial superoxide surge is the ETC, likely due to excessive reduction in the coenzyme Q pool and elevated membrane potential following ATP synthase blockade. This leads to reverse electron transport and enhanced superoxide generation, which, coupled with compromised antioxidant systems, triggers profound mitochondrial oxidative stress [4]. Mitochondrial enzymatic Complex II has shown greater sensitivity to direct NAPQI inhibition in vitro, while in vivo models demonstrate that APAP overdose significantly suppresses Complex I activity [38]. Furthermore, APAP interferes with the formation of respiratory supercomplexes via the negative regulator methylation-controlled J protein (MCJ), reducing ATP production and increasing ROS [41,42,43].

At high APAP doses, thiol group modification induces an immediate halt in mitochondrial respiration. The inhibition of Complexes I and II facilitates an electron leak that reduces molecular oxygen to superoxide radicals (O_2_^−•^), potent ROS and damaging factor [15]. Under GSH deficiency, superoxide reacts with nitric oxide (NO^•^) to form peroxynitrite (ONOO−), highly aggressive nitrosating agent that causes tyrosine nitration, organelle collapse, and mtDNA damage [12]. Peroxynitrite in the mitochondrial matrix is currently viewed as the critical oxidant responsible for triggering the opening of the mitochondrial permeability transition pore (mPTP) [44]. Nitrotyrosine protein adducts were also detected within mitochondria, together with evidence of a specific elevation in mitochondrial GSSG levels [45].

Many AMPs are capable of interacting not only with bacterial membranes but also with mitochondria, whose membranes share important structural and physicochemical similarities with prokaryotic membranes. In particular, several AMPs exhibit affinity toward cardiolipin-rich mitochondrial membranes, where they may induce membrane permeabilization, dissipation of mitochondrial membrane potential, oxidative stress, and alterations in ATP production [46,47]. Some AMPs can additionally translocate into cells and directly affect mitochondrial integrity and function through intracellular mechanisms [48,49]. Since mitochondrial dysfunction and membrane destabilization are central events in APAP-induced toxicity, potential interactions between APAP and AMPs at the mitochondrial level warrant further investigation.

The role of LPO in APAP-induced hepatotoxicity has been described, although the extent of its contribution to liver injury remains insufficiently defined. Current evidence suggests that LPO represents a secondary pathogenic process that develops following the direct toxic effects of APAP metabolism and NAPQI formation, rather than the primary mechanism of cell death. Nevertheless, the protective effects observed with mitochondrial aldehyde dehydrogenase 2 (ALDH2) inducers indicate that localized mitochondrial LPO contributes to the progression of injury [50]. The accumulation of lipid- and protein-derived aldehydes, together with impaired aldehyde detoxification pathways, may further aggravate oxidative damage during toxic hepatosis. In particular, 4-hydroxynonenal (4-HNE), a major end-product of LPO capable of modifying functional proteins, enzymes, and receptors [51,52], is a plausible mediator linking oxidative stress to impaired mitochondrial function. Accordingly, an imbalance between the generation of reactive aldehydes and their enzymatic degradation is likely to exacerbate hepatocellular damage [53]. Furthermore, localized LPO may act synergistically with peroxynitrite to amplify oxidative injury [54]. However, the observation that several antioxidant and free radical–targeting interventions attenuate, but do not completely prevent, APAP-induced liver injury supports the view that LPO primarily amplifies the toxic cascade rather than initiating it.

Interestingly, MnSOD is a target for peroxynitrite modification, further impairing the cell’s ability to handle superoxide [55]. The significance of peroxynitrite is supported by experiments showing that SOD2 deficiency exacerbates liver injury, whereas mitochondrial-targeted SOD mimetics such as Mito-TEMPO markedly reduce peroxynitrite formation and attenuate hepatotoxicity [38,56]. Furthermore, the genetic deficiency of neuronal nitric oxide synthase (nNOS) or the administration of nNOS inhibitors has been shown to attenuate APAP-induced hepatic injury and diminish nitrotyrosine staining [55]. Collectively, these findings indicate that peroxynitrite serves as an important mediator in the pathophysiology of APAP-induced hepatotoxicity, promoting the degradation of mitochondrial proteins, such as MnSOD, and causing mitochondrial DNA (mtDNA) damage, without necessarily inducing concomitant LPO [54].

In another study, during APAP overdose, mitochondrial oxidative stress initiates cytosolic signaling cascades that amplify organelle damage. Despite its short half-life, peroxynitrite can traverse biological membranes and diffuse across cell diameters [57]. Evidence suggests that peroxynitrite is capable of traversing erythrocyte membranes either via anion channels in its anionic state or through passive diffusion in its protonated form. This implies that APAP-induced peroxynitrite, synthesized within the mitochondria, may either passively diffuse or be actively transported across mitochondrial anion channels, such as the voltage-dependent anion channel (VDAC), which regulates the efflux of superoxide anions from the mitochondria into the cytosol [4]. Elevated cytosolic peroxynitrite levels trigger the activation of the mitogen-activated protein kinase (MAPK) cascade, culminating in the activation of c-Jun N-terminal kinase (JNK), which subsequently exacerbates mitochondrial dysfunction [4].

A pivotal step in APAP-induced signal transduction is the cytosolic activation of c-Jun N-terminal kinase (JNK). Oxidative stress activates a MAPK cascade leading to JNK phosphorylation (p-JNK) [58]. Phosphorylated JNK translocates to the outer mitochondrial membrane, where it binds to Sab and triggers intramitochondrial signaling that inhibits the respiratory chain, amplifying ROS production in a self-sustaining cycle [59]. The critical role of mitochondrial JNK translocation is supported by the cytoprotection observed when this process is pharmacologically or genetically inhibited [60]. Sustained oxidative stress and protein nitration ultimately induce opening of the mitochondrial permeability transition pore (mPTP), leading to loss of membrane potential (ΔΨm), ATP depletion, mitochondrial swelling, and release of intermembrane proteins such as cytochrome c, EndoG, and AIF, culminating in irreversible cell death [15,58,61]. Additionally, lysosomal iron translocation occurs in hepatocytes following APAP treatment, contributing to mPTP induction [62]. This iron translocation is mediated by the mitochondrial calcium uniporter [63] and is likely linked to lysosomal instability, a phenomenon also observed following APAP overdose. While the efflux of cytochrome c disrupts the mitochondrial electron transport chain and terminates ATP synthesis, both apoptosis-inducing factor (AIF) and endonuclease G possess nuclear localization signals (NLS) that facilitate their subsequent translocation to the nucleus, where they trigger chromatin condensation and DNA fragmentation [4].

Note, in contrast to classical apoptosis, the ATP depletion during APAP poisoning could prevent caspase activation; thus, cell death could proceed via oncotic necrosis [64]. Released intermembrane proteins like AIF and EndoG drive nuclear DNA fragmentation [64], whereas cytochrome c and Smac/DIABLO do not contribute to apoptosis because the biochemical environment prevents such signaling [65]. It has also been demonstrated that, in hepatocytes surrounding necrotic regions, mitochondrial function can be restored through mitochondrial biogenesis, resulting in a progressive recovery of electron transport chain activity within 24–72 h following APAP administration. In addition to biogenesis, mitochondrial function may also be re-established through spontaneous repolarization in surviving hepatocytes. Notably, mitochondrial repolarization occurs significantly earlier than biogenesis and is largely completed within 24 h after APAP exposure [66].

Given that mitochondria are highly dynamic organelles undergoing continuous fusion and fission to preserve cellular homeostasis, increasing attention has been directed toward the impact of APAP overdose on mitochondrial dynamics. Early evidence demonstrated a marked upregulation of the fission mediator dynamin-related protein 1 (Drp1) and its translocation to mitochondria following APAP exposure [67]. These findings suggest that APAP-induced alterations in mitochondrial dynamics may contribute to the modulation of intracellular signaling pathways and influence the progression of hepatocellular injury.

In summary, both protein adduct formation and the induction of oxidative stress, including lipid peroxidation, represent hallmark features of APAP-mediated cell death and are mechanistically interdependent. Mitochondria-associated oxidative stress is considered a critical driver of injury progression. In this context, potential interactions with AMPs, many of which are capable of modulating membrane integrity, mitochondrial function, and redox homeostasis, may represent an additional and currently underexplored factor influencing APAP-induced toxicity.

### 2.3. Effects of Acetaminophen on the Plasma Membrane and Ionic Homeostasis

Beyond intracellular organelles, acetaminophen in high concentrations and its metabolites exert a destructive influence on the plasma membrane. Hepatocellular disintegration following acetaminophen overdose is driven by the loss of plasma membrane selective permeability and the collapse of cellular architecture. This necrotic progression causes a massive efflux of organ-specific enzymes into the systemic circulation, often appearing alongside a marked retention of biliary metabolites [68]. An early sign of hepatotoxicity is the 52% reduction in Na^+^/K^+^-ATPase activity—the primary enzyme maintaining the electrochemical gradient. This decline occurs within 3 h of APAP toxic exposure, preceding the release of ALT into the blood [69]. This inhibition is tied to the direct action of NAPQI, which oxidizes critical thiol groups to form disulfide bridges, blocking the conformational changes necessary for ion transport and leading to osmotic swelling and lysis.

Acetaminophen overdose triggers an uncontrolled increase in cytosolic calcium. TRPM2 channels (Transient Receptor Potential Melastatin 2), activated by ROS and mitochondrial damage products, play a pivotal role in this process [70]. Elevated APAP levels promote extracellular Ca^2+^ influx, leading to calpain activation and degradation of cytoskeletal and membrane-anchor proteins, thereby further aggravating ionic imbalance [71]. where marked alterations in plasma and hepatic phospholipid profiles, likely reflecting both direct damage and disrupted phospholipid metabolism [72]. Although LPO contributes to oxidative membrane damage, current evidence suggests that it primarily amplifies injury initiated by NAPQI-induced mitochondrial dysfunction rather than acting as the primary mechanism of membrane disruption. In this context, oxidation of polyunsaturated fatty acids has been proposed to contribute to ferroptotic signaling during APAP hepatotoxicity [73]. However, the available data do not establish ferroptosis as the dominant mechanism of cell death, but rather suggest that it may represent one component of the complex network of oxidative injury. In parallel, marked alterations in plasma and hepatic phospholipid profiles have been reported following APAP exposure, likely reflecting both membrane damage and disrupted phospholipid metabolism [72].

Another study studied involvement of iron in APAP-induced hepatotoxicity with possible consequences for plasmatic membranes. It is now recognized that APAP precipitates lysosomal instability [74], triggering the release of iron from this compartment and subsequently elevating cytosolic ferrous iron (Fe^2+^) levels. This iron is sequestered by the mitochondria via the mitochondrial electrogenic Ca^2+^/Fe^2+^ uniporter (MCFU). Once inside the mitochondrial matrix, iron facilitates the opening of the mPTP and subsequent cell death, a mechanism supported by the protective effects of lysosomal iron chelators and MCFU inhibitors [63].

Furthermore, experimental activation of the protective enzyme aldehyde dehydrogenase reduces the levels of 4-HNE [50], widely associated with cellular membrane damage in various pathological conditions [52,75,76]. Notably, 4-HNE may act both intracellularly and by diffusing from its site of production to adjacent cells [52,77,78], thereby propagating oxidative damage and protein modifications.

The aldehyde dehydrogenase partially attenuates mitochondrial permeability transition pore (mPTP) opening and subsequent cell death [50]. Collectively, these findings suggest a complex interplay between iron mobilization, localized mitochondrial LPO, and the potential involvement of multiple cellular membranes in the pathophysiology of APAP-mediated cell death.

The potential interaction of APAP with cardiolipin is of particular interest due to the essential role of this phospholipid in mitochondrial membrane integrity [79]. APAP has been reported to penetrate lipid membranes and, at therapeutic concentrations, can prevent hemoprotein-catalyzed lipid peroxidation both in vitro and in vivo by reducing ferryl heme to its ferric state and inhibiting cytochrome c redox cycling, thereby limiting cardiolipin oxidation [80]. In contrast, at high doses, APAP induces mitochondrial dysfunction and disrupts cardiolipin homeostasis. Recent evidence demonstrates that APAP overdose is associated with cardiolipin pathway modulation, and that inhibition of neddylation in APAP-induced liver injury restores cardiolipin levels, highlighting the importance of cardiolipin in mitochondrial dysfunction during APAP toxicity [79].

Interestingly, cardiolipin has been proposed as a component of liposomal carriers for the antimicrobial peptide gramicidin S [81,82]. Consequently, APAP-induced alterations of cardiolipin and mitochondrial membrane organization may represent an important factor to consider in the formulation and stability of such liposomal delivery systems.

As mentioned above, acetaminophen itself can directly integrate into the lipid bilayer. The effects of high-dose APAP were studied on pure phospholipid membranes. Using 1,2-di-(octadecenoyl)-sn-glycero-3-phosphocholine (DOPC) large unilamellar vesicles (LUVs) and a combination of dynamic light scattering (DLS), small-angle neutron scattering (SANS), small-angle X-ray scattering (SAXS), cryogenic transmission electron microscopy (cryo-TEM), and neutron spin-echo (NSE) spectroscopy, it was shown that APAP incorporation (0.06–0.12 wt%) significantly alters vesicle morphology and lipid organization, reducing the lipid number per vesicle (by ~28% and ~19%, respectively), decreasing membrane bending rigidity by ~50%, and enhancing lipid tail dynamics (up to 1.75-fold), ultimately leading to more irregular and mechanically softer membranes despite minimal changes in bilayer thickness [72]. Another study investigated the interaction of acetaminophen with biomimetic lipid bilayer membranes mimicking neuronal, hepatic, and renal cells and compared it with phenacetin, a structurally related para-aminophenol derivative, finding that although both drugs increased membrane fluidity in a lipid composition-dependent manner, acetaminophen’s membrane interactivity was specifically associated with nephrotoxicity [83]. The known impact of APAP/NAPQI on membrane parameters is summarized in Table 2.

These findings suggest that, in living cells, such biophysical alterations may impair not only lipid organization and membrane properties but also the activity of membrane-associated proteins, (e.g., COX), thereby reducing cellular resistance to mechanical and osmotic stress. In addition, these alterations could influence interactions with membrane-targeting antibiotics, including AMPs.

### 2.4. Toxicological Characteristics of Acetaminophen Under Protein Deficiency with a Focus on Membrane Targeting

Nutritional status is a critical determinant of individual sensitivity to acetaminophen. Dietary protein deficiency dramatically lowers the drug’s tolerance, making even therapeutic doses potentially lethal [21]. Several mechanisms drive this increased sensitivity during protein starvation. Primary among these is the critical reduction in the glutathione pool. GSH synthesis depends also on sulfur-containing amino acids (cysteine and methionine), the limited supply of which during protein deficiency can lower baseline hepatic GSH by 40–80%. Consequently, the threshold for exhausting antioxidant defenses is reached much sooner than in standard nutrition.

Interestingly, short-term starvation for three days has been reported to double renal CYP2E1 levels in rats, suggesting an immediate inductive effect of acute nutrient deprivation. In contrast, long-term exposure to a low-calorie, low-protein diet for one month results in decreased CYP2E1 expression [84], possibly reflecting adaptive mechanisms aimed at reducing potentially harmful CYP enzyme activity [85] relatively little. However, given the extensive localization of CYPs in the endoplasmic reticulum, membrane-related effects could potentially affect CYP structure and function.

Furthermore, under conditions of protein deficiency, APAP intoxication results in a profound suppression of mitochondrial NAD+-dependent dehydrogenases, including isocitrate dehydrogenase and α-ketoglutarate dehydrogenase, leading to a near-total collapse of aerobic respiration. Also, this study has demonstrated that dietary protein restriction critically exacerbates mitochondrial dysfunction in both the kidneys and liver following APAP administration. This state is characterized by significant inhibition of the mitochondrial respiratory chain, specifically involving the activities of Complex I (NADH dehydrogenase) and Complex II (succinate dehydrogenase) [86].

This bioenergetic crisis is characterized by severe disruption of the adenylate nucleotide system. A combination of protein restriction and APAP-induced toxemia leads to marked physiological decline and organ-specific damage, including reduced body, liver, and kidney mass [43]. In renal mitochondria exposed to supratherapeutic APAP doses, ATP levels drop sharply, accompanied by increased ADP and AMP concentrations. This imbalance activates AMP deaminase and ATPases, while markedly suppressing cytosolic 5′-nucleotidase activity, indicating profound nucleotide dysregulation [87]. AMP deaminase activation reflects failure of energy homeostasis and contributes to plasma membrane instability through impaired ion pump function [87]. Studies demonstrate that rats on a 4% protein diet show much higher mortality and larger necrotic zones at 800 mg/kg APAP compared to those on normal 15–20% protein diets. Supplementing the diet with glycine or cysteine can partially restore resistance, highlighting the importance of substrate availability [88]. The factors reported to influence APAP toxicity under conditions of protein malnutrition are summarized in Table 3. Although the direct involvement of cell membranes has not yet been investigated under conditions of malnutrition, the membrane-related effects described in APAP toxicity are likely to be amplified during nutrient deficiency and therefore warrant further investigation.

### 2.5. Immunological Mechanisms in Acetaminophen Toxicity

The role of the immune response in APAP toxicity has been investigated; however, it remains incompletely understood, and the available data are partially conflicting [5,18,92]. APAP-induced liver injury is closely linked to inflammation, which is initiated by hepatocyte necrosis following excessive APAP metabolism and subsequently shaped by immune cell recruitment and cytokine release. Importantly, inflammatory responses in APAP hepatotoxicity exert a dual role, contributing both to the amplification of liver injury and to the resolution of tissue damage by promoting liver regeneration and repair [5,92].

APAP overdose triggers hepatocyte necrosis and a concomitant sterile inflammatory response involving innate immune cells, including neutrophils and monocytes/macrophages. In addition to intracellular damage, APAP-induced membrane perturbation, lipid peroxidation, and alterations in membrane organization may contribute to damage-associated molecular patterns (DAMPs) release and immune-cell activation. Through the release of DAMPs, antigen-presenting cells (APCs), such as dendritic cells (DCs), are also engaged [5]. For example, following APAP overdose in mice, hepatic dendritic cells exhibit an altered immune phenotype, including increased expression of MHC II and co-stimulatory molecules, along with elevated production of pro-inflammatory cytokines (IL-6, MCP-1, and TNF-α), indicating phenotypic activation of DCs [93]. Note, in this study depletion of DCs exacerbates liver injury, indicating that DC activation is not exclusively pro-inflammatory but also contributes to limiting tissue damage and promoting the resolution of inflammation [93], despite the general consideration of overall detrimental impact of excessive innate immune activation in APAP-induced liver injury.

Potential alterations in immune cell phenotype, maturation, or functional processes during acetaminophen toxicity may resemble those described in other pathological conditions, where (i) membrane damage and lipid peroxidation [94,95,96,97], (ii) chromatin remodeling [98,99], and (iii) functional impairment associated with cytoskeletal disorganization [100,101] have been shown to play mechanistic roles.

APAP-induced oxidative stress and lipid peroxidation are likely to influence immune responses by promoting membrane damage, the formation of oxidized lipids and bioactive lipid peroxidation products, the release of DAMPs, and the activation of innate inflammatory pathways [3,37,73]. For example, lipid peroxidation products such as 4-hydroxynonenal (4-HNE) and the oxidized arachidonic acid derivative 15-hydroxyeicosatetraenoic acid (15-HETE), a ligand of the nuclear receptor PPARγ, have been shown to possess immunomodulatory activity [102], whereas acetaminophen-induced liver injury strongly interferes with hepatic lipid metabolism and eicosanoid signaling pathways [103].

Importantly, the majority of mechanistic insights derive from rodent models; therefore, caution is warranted when extrapolating these findings to human systems. Despite their extensive and longstanding use, significant immunological differences between rodents and humans have been documented, including species-specific immune cell distributions, receptor expression patterns, signaling cascades, and inflammatory responses [104,105,106]. These interspecies differences may substantially influence the magnitude, kinetics, and resolution of immune activation during APAP-induced injury. Consequently, further studies employing human-relevant models are required to clarify immune cell–specific mechanisms, delineate context-dependent pro- and anti-inflammatory pathways, and establish the translational relevance of immune modulation in acetaminophen toxicity.

## 3. Mechanisms of Interaction of Antimicrobial Peptides with Cell Membranes and Model Lipid Membrane Systems

### 3.1. Biophysical Principles Governing Antimicrobial Peptide—Membrane Interactions

In contrast to APAP, the membrane and cellular effects of AMPs are generally independent of their biochemical modification, as AMPs are only rarely involved in host–cell metabolic or catabolic pathways. Instead, their activity arises primarily from noncovalent interactions with membranes and intracellular targets. These interactions are governed mainly by electrostatic and hydrophobic forces and may result in membrane binding, lipid redistribution, alterations in membrane fluidity and organization, pore formation, transient permeability defects, or membrane translocation followed by intracellular targeting [107,108,109].

The biological activity of membrane-active peptides therefore depends on their ability either to perturb membrane integrity and function or to cross the lipid bilayer and modulate intracellular processes [47,48,49]. The outcome of these interactions is determined by peptide structure, membrane composition, and physicochemical conditions. Because of their complexity, peptide–membrane interactions are increasingly investigated using complementary experimental and computational biophysical approaches, which are also supporting the development of membrane-based therapeutics and delivery systems [107,110,111].

Among membrane-active peptides, AMPs constitute one of the most extensively studied and therapeutically relevant groups. These molecules exert activity against a broad spectrum of pathogens, including bacteria, fungi, and viruses, through mechanisms involving either membrane disruption or interference with essential cellular pathways [108,109]. The accelerating spread of antimicrobial resistance has progressively reduced the efficacy of many conventional antibiotics, thereby intensifying the search for alternative anti-infective strategies. In this context, naturally occurring AMPs, which serve as key effectors of innate immune defense, have attracted considerable attention as prospective therapeutic agents due to their broad-spectrum activity, mechanistic diversity, and reduced susceptibility to classical antibiotic resistance mechanisms [112].

A distinguishing feature of membrane-active peptides is their ability to selectively interact with cellular surfaces that differ in lipid composition, charge, and structural organization. Rather than acting through a single molecular target, many peptides engage multiple membrane-associated components and supramolecular assemblies, resulting in pleiotropic biological effects [49,108]. Their activity is further modulated by the local microenvironment, as factors such as pH, ionic strength, lipid composition, and lipid oxidation can markedly influence peptide binding, membrane insertion, and subsequent biological responses [108,110,113].

Membrane-active peptides encompass a structurally heterogeneous group of molecules whose biological behavior is nevertheless frequently associated with two common physicochemical features: amphipathicity and an overall positive charge [108,109]. Despite sharing these general characteristics, their amino acid composition, sequence organization, and three-dimensional conformations display substantial variability. Unlike highly specific receptor-targeted therapeutics, AMPs have evolved as multifunctional defense molecules capable of interacting with diverse biological targets rather than a single molecular structure. This broad target spectrum gives rise to a variety of biological effects and contributes to their ability to evade conventional microbial resistance mechanisms [112].

Peptide–membrane interactions are strongly influenced by amino acid composition and sequence, which determine charge distribution, hydrophobicity, and conformational flexibility, together with the lipid organization of the target membrane. In addition, membrane binding and structural perturbations are often concentration-dependent, reflecting local peptide accumulation at the membrane interface [114]. Consequently, the same peptide may exhibit distinct membrane-associated behaviors under different conditions, including variations in bilayer composition or peptide density, ultimately influencing the extent and mode of membrane destabilization [115].

The physicochemical characteristics of lipid bilayers further determine peptide susceptibility by controlling membrane phase behavior, lateral organization, and lipid packing properties [116]. Such membrane-dependent heterogeneity substantially modulates AMP activity and may influence the balance between membrane permeabilization and non-lytic mechanisms of action. Moreover, in organisms possessing a cell wall, or in cells with developed glycocalyx, its structural organization contributes to regulating peptide accessibility to the plasma membrane by affecting local peptide accumulation near the membrane surface, thereby shaping the subsequent interaction mechanism [117,118].

The biological consequences of AMP–cell interactions are highly diverse and extend beyond membrane disruption. Some AMPs interfere with essential surface-associated processes, for example by binding membrane-associated precursors such as lipid II and lipid A, thereby inhibiting bacterial cell wall biosynthesis [119]. Others compromise membrane function through permeabilization mechanisms, including pore formation and carpet-like disruption driven by peptide accumulation at the membrane interface [46] (see also Section 3.3.1 and Section 3.3.2). However, membrane permeabilization is not a universal outcome of AMP activity. Certain peptides induce reversible changes in membrane organization, such as redistribution of membrane-associated proteins via interactions with lipid microdomains [120], whereas others translocate across lipid bilayers with limited membrane damage and subsequently modulate intracellular metabolic and regulatory pathways [48].

Membrane-active peptides are frequently categorized according to their predominant secondary structural motifs, which include α-helical, β-structured, mixed α/β, and intrinsically disordered conformations [121]. Nevertheless, such classification schemes should be regarded as context-dependent rather than absolute, since peptide conformation is highly sensitive to environmental conditions and may undergo substantial rearrangements in response to changes in membrane composition, local peptide concentration, or surrounding physicochemical parameters. Peptide–membrane interactions therefore represent dynamic adaptive processes in which both the peptide structure and the lipid environment mutually influence one another. Importantly, secondary structure stabilization in lipid bilayers is governed by principles distinct from those in aqueous solution, with hydrogen bonding and low dielectric permittivity playing central roles in maintaining peptide conformations and supramolecular assemblies [122].

Certain classes of membrane-active peptides exhibit relatively well-defined conformational preferences in solution prior to membrane association. In particular, cyclic peptides and peptides stabilized by intramolecular disulfide bridges often adopt compact and ordered structures in aqueous environments, although membrane insertion may induce pronounced conformational reorganization. A representative example is PG-1, which forms oligomeric transmembrane β-barrel assemblies in anionic membranes mimicking bacterial lipid composition, whereas in cholesterol-enriched bilayers representative of eukaryotic membranes, the peptide preferentially adopts surface-associated β-sheet aggregates localized at the membrane interface [123].

### 3.2. AMP–Membrane Complexation and Peptide Oligomerization

The amino acid composition of membrane-active peptides is a key determinant of their structural behavior, influencing secondary structure, intermolecular association, and aggregate formation. Together with physicochemical properties such as hydrophobicity and net charge, these features strongly affect antimicrobial potency and membrane activity. However, peptide conformation and aggregation are highly context-dependent rather than fixed intrinsic properties. Many linear peptides exhibit substantial conformational plasticity, adopting different structural states in aqueous and membrane-associated environments. Amyloidogenic peptides provide an illustrative example, as they readily form β-sheet-rich supramolecular assemblies while sharing several structural and functional characteristics with AMPs [124]. These similarities suggest that aggregation-prone structural motifs may play an important role in membrane activity and biological function.

Peptide–lipid complex formation is a fundamental aspect of AMP activity. Beyond membrane binding, peptide self-assembly can profoundly influence membrane architecture, permeability, and function [125]. Aggregation is driven by a combination of electrostatic, hydrogen-bonding, hydrophobic, and π–π interactions that determine the stability and morphology of the resulting supramolecular structures [126]. Importantly, these processes are highly sensitive to environmental conditions, with ionic strength, pH, and solvent polarity acting as major modulators of self-assembly behavior [127].

Because membrane-active peptides encounter their targets through aqueous environments, their behavior in electrolyte solutions is highly relevant to biological activity. Peptide self-association prior to membrane binding can markedly influence antimicrobial efficacy, with excessive aggregation often correlating with reduced activity, likely due to diminished membrane accessibility [128,129,130]. Conversely, increasing peptide cationicity has been reported to suppress aggregation and enhance antimicrobial potency [131].

Aggregation propensity is determined by both primary sequence and conformational constraints. Structural stabilization through cyclization or intramolecular covalent linkages can alter peptide dynamics and self-assembly in aqueous and membrane environments [128]. In cationic peptides, aggregation is often governed by electrostatic interactions, while differences in dielectric permittivity between aqueous and lipid phases further modulate intermolecular forces and supramolecular organization. Notably, several of these factors may be altered during APAP-induced toxicity, where reactive metabolites and membrane perturbations could influence AMP conformation, self-assembly, membrane affinity, and biological activity.

The link between peptide self-assembly and antimicrobial activity is exemplified by the self-assembling dipeptide diphenylalanine [132]. As a key recognition motif of β-amyloid peptides, it demonstrates that aggregation can directly contribute to biological function. Diphenylalanine assemblies disrupt bacterial membranes and inhibit microbial growth, whereas non-assembled peptide displays only limited antibacterial activity. Its hydrophobic, non-cationic character further makes it a promising scaffold for overcoming resistance mechanisms that weaken interactions between microbial membranes and cationic AMPs.

In addition to the biophysical factors discussed above, aromatic π–π interactions can contribute significantly to the stability and organization of supramolecular peptide assemblies [132]. However, aromatic residues do not necessarily promote aggregation more effectively than non-aromatic amino acids of comparable hydrophobicity. Rather, they often influence the morphology and structural organization of peptide aggregates, including the formation of distinct fibrillar architectures [126].

Advances in the understanding of peptide self-assembly mechanisms are increasingly being translated into the development of antimicrobial nanomaterials, opening new opportunities for addressing microbial resistance [133]. Membrane-active peptides, enriched in hydrophobic, aromatic, and charged residues, readily organize into supramolecular structures, such as amyloid-like fibrils or helical bundles, within membrane-associated environments. Such architectures strengthen interactions with bacterial membranes and may substantially improve antimicrobial performance [134]. Furthermore, self-assembled peptide nanostructures can prolong peptide stability and increase therapeutic persistence, while simultaneously enabling spatial control over local peptide concentration, thereby enhancing membrane interactions and targeting efficiency [133].

In addition to charge distribution, hydrophobicity, and secondary structure, amino acid chirality is an important determinant of AMP activity. The enhanced antimicrobial efficacy often observed for D-enantiomeric peptides cannot be attributed solely to increased resistance to proteolytic degradation. Notably, heterochiral peptides containing both D- and L-amino acid residues frequently exhibit a greater propensity for self-assembly into stable supramolecular structures and enhanced membrane-permeabilizing activity compared with homochiral analogues [135]. These observations suggest that chirality influences peptide–membrane interactions through its effects on supramolecular organization, thereby modulating biological activity. They also highlight the importance of the membrane physicochemical environment in AMP function and may provide insight into off-target interactions with mammalian membranes. However, the contribution of self-assembly is not universal, as some AMPs retain substantial antimicrobial activity irrespective of their aggregation state.

Collectively, these findings indicate that AMP activity is governed by a complex interplay between peptide-specific molecular features and membrane physicochemical properties, suggesting that other membrane-active compounds may modulate AMP behavior. Within this context, APAP, although predominantly distributed in the aqueous phase, has been reported to interact directly with lipid bilayers and alter membrane morphology, lipid packing, and lipid dynamics [72]. Such effects could, according to our hypothesis, influence AMP activity.

Given the strong dependence of AMP function on membrane organization, APAP-induced membrane remodeling may affect peptide binding, insertion, self-assembly, translocation, and pore formation. Alterations in membrane fluidity, lipid packing, dielectric properties, and interfacial organization could modify peptide aggregation thresholds and membrane crowding effects, thereby changing AMP efficacy and potentially promoting cooperative interactions at the membrane level [136,137]. Because supramolecular assembly often regulates AMP function, these effects may also extend to peptide aggregation in the aqueous phase, facilitating drug–peptide interactions before membrane contact. However, these mechanisms remain hypothetical and require systematic experimental and computational validation.

### 3.3. Putative Models of AMP Interaction with Biological Membranes

AMPs may exert their biological effects through distinct membrane-associated pathways, including direct permeabilization of lipid bilayers or membrane translocation followed by interactions with intracellular targets in the absence of immediate membrane disruption [108]. The balance between these mechanistic outcomes depends on multiple peptide- and membrane-related factors, including peptide structure, membrane composition, and local physicochemical conditions.

Considerable advances in understanding peptide–membrane interactions have been achieved through the combined use of computational simulations and experimental biophysical approaches. For example, free-energy profiles of membrane insertion for the model tryptophan/leucine hexapeptide WL_5_ revealed multiple energetically favorable conformational states at different depths within the lipid bilayer [138]. A metastable minimum is commonly observed near the water–lipid interface, particularly within the phospholipid headgroup region, whereas a more favorable energetic state is located deeper in the membrane, near the glycerol backbone at the hydrophilic–hydrophobic boundary [81,111]. These findings indicate that peptide localization within the bilayer is governed by multiple energetically accessible states. For many AMPs, the energetic barrier for translocation across the hydrophobic core is relatively low (~1 kcal/mol), possibly reflecting increased conformational flexibility upon membrane insertion. In addition, peptide orientation and preferential partitioning into specific membrane microdomains can substantially influence insertion energetics and promote alternative pathways of membrane association [111].

Computational studies indicate that peptide translocation across lipid membranes cannot be described solely by insertion depth or peptide position within the bilayer. Instead, membrane remodeling, local bilayer deformation, and conformational changes in charged peptide groups all contribute to the energetics of membrane crossing. Coarse-grained molecular simulations have shown that accurate prediction of peptide translocation requires simultaneous consideration of peptide insertion, membrane deformation, and peptide conformational dynamics [136]. Within the framework of the present review, these findings suggest that APAP-induced changes in membrane organization and mechanics may alter the free-energy landscape governing AMP insertion and translocation.

#### 3.3.1. Membrane Permeabilization Strategies for AMPs

Membrane permeabilization represents one of the most intensively investigated manifestations of AMP activity and has been explored through a broad spectrum of experimental and theoretical approaches [139,140,141]. Experimental characterization is commonly performed using model membrane systems, including large unilamellar vesicles (LUVs) and giant unilamellar vesicles (GUVs), where membrane leakage assays are frequently employed to quantify peptide-induced disruption [142,143,144,145]. AMP-mediated membrane permeabilization is commonly classified into three principal mechanisms: barrel-stave pore formation, toroidal pore formation, and carpet-like membrane disruption. However, the precise mode of action remains strongly dependent on both peptide and membrane properties [146,147].

Despite sharing the common outcome of increased membrane permeability, these mechanisms differ substantially in the molecular organization of peptides and lipids during membrane disruption. In the barrel-stave model, amphipathic peptides insert deeply into the lipid bilayer and subsequently oligomerize into transmembrane assemblies, generating well-defined pores in which the hydrophilic peptide surfaces line the aqueous lumen, whereas hydrophobic residues remain oriented toward lipid acyl chains [148]. This mechanism is generally associated with structurally ordered peptides capable of forming stable α-helical or β-sheet conformations and often requires relatively rigid peptide architectures [149]. In contrast, toroidal pore formation involves a cooperative contribution of both peptides and membrane lipids, resulting in curved pore structures where the lipid monolayers bend continuously through the pore and lipid headgroups actively participate in channel formation [149,150]. Electrostatic interactions between cationic peptides and negatively charged phospholipid headgroups frequently stabilize such structures, making toroidal pores particularly relevant in bacterial membranes enriched in anionic lipids. By comparison, the carpet mechanism does not necessarily involve formation of stable transmembrane pores. Instead, peptides accumulate at the membrane interface in a surfactant-like manner until a threshold surface concentration is reached, after which large-scale membrane destabilization and transient rupture occur without the establishment of persistent, structurally defined channels [151]. Collectively, these models indicate that AMP-induced membrane perturbation spans a continuum ranging from highly ordered pore architectures to more stochastic bilayer-disruptive processes. The prevailing mechanism depends on peptide structure, membrane composition, and local physicochemical conditions. Within the framework of the present hypothesis, APAP-induced changes in membrane properties may further modulate these processes, potentially affecting peptide partitioning, oligomerization, translocation, and overall biological activity.

#### 3.3.2. Membrane Destabilization by AMPs

AMP-induced membrane destabilization encompasses processes ranging from local membrane thinning and transient lipid defects to extensive bilayer disruption. At sufficiently high concentrations, amphipathic peptides may also oligomerize in solution prior to membrane binding, thereby influencing the kinetics and energetics of peptide–membrane interactions. This behavior is commonly described by the two-state model, in which peptides dynamically partition between a surface-bound state and a membrane-inserted state capable of inducing transmembrane defects and pore formation [115].

In parallel with membrane-disruptive activity, AMPs may exert biological effects through non-lytic mechanisms that preserve membrane integrity to a substantial extent [152]. These alternative pathways include peptide translocation across lipid bilayers without extensive membrane damage [49], modulation of intracellular molecular targets [47], and interference with essential metabolic processes [48,153]. Such non-lytic modes of action are considered particularly relevant for antiviral and anticancer peptides, where selective intracellular targeting may occur in the absence of overt membrane destruction [47].

AMP activity arises from a dynamic interplay between peptide properties and membrane physicochemical characteristics rather than a single universal mechanism [47,154]. As a result, the same peptide may operate through multiple mechanisms or transition between different modes of action depending on membrane properties. Thus, APAP may represent a previously underappreciated modulator of AMP–membrane interactions. By altering membrane organization and local physicochemical parameters, APAP could indirectly reshape the free-energy landscape governing peptide insertion and membrane destabilization. In particular, APAP-mediated perturbations of membrane polarity gradients, lipid packing density, and interfacial organization may influence energetic barriers associated with peptide insertion, alter insertion depth, and modify peptide orientation within the bilayer. Although such mechanisms remain largely hypothetical, they provide a plausible physicochemical basis through which APAP may modulate AMP membranotropic activity and therefore warrant systematic investigation.

**Figure 2:**
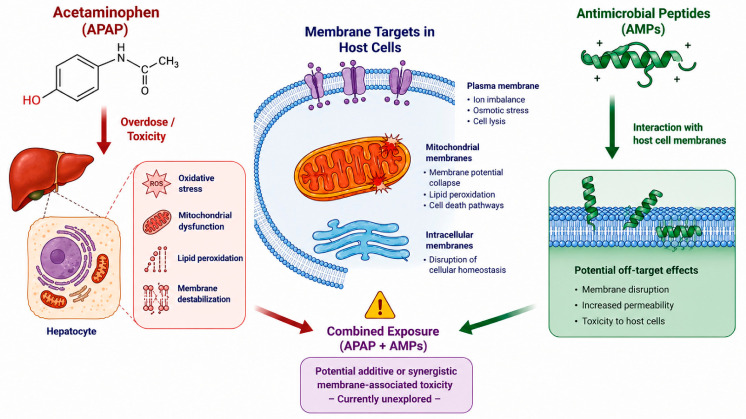
Schematic representation of the membrane-targeted consequences of acetaminophen (APAP) toxicity and the potential off-target effects of antimicrobial peptides (AMPs) on host cell membranes. APAP overdose is associated with oxidative stress, mitochondrial dysfunction, lipid peroxidation, and membrane destabilization, affecting plasma, mitochondrial, and intracellular membranes. AMPs, while primarily targeting microbial membranes, may also interact with host cell membranes, leading to increased permeability and membrane-associated toxicity. The figure further highlights the currently unexplored possibility of additive or synergistic toxic effects during combined exposure to APAP and AMPs. The figure was generated with the assistance of an AI-based image generation tool (GPT-5.3). The authors validated all scientific content and take full responsibility for the accuracy and integrity of the figure.

Beyond potential effects on insertion energetics, APAP-induced membrane remodeling may also affect downstream stages of membrane destabilization. Evidence from hepatotoxicity-associated membrane injury indicates that APAP can alter membrane permeability and contribute to ion imbalance (see Section 2.3). Furthermore, oxidative membrane damage, lipid peroxidation, and bilayer disordering induced by APAP may create membrane conditions that facilitate AMP-mediated permeabilization, potentially enhancing membrane leakage or lowering thresholds required for pore formation. Such cooperative effects could substantially modify membranolytic activity and may represent an overlooked contributor to host–cell membrane vulnerability under conditions of combined APAP and AMP exposure, as conceptually illustrated in Figure 2.

An additional consequence of APAP-mediated membrane remodeling may be a shift in the dominant mode of AMP action. Changes in membrane physicochemical properties induced by APAP could theoretically favor transitions between different membrane interaction pathways, for example shifting peptide activity from surface-associated carpet-like perturbation toward stable pore formation, or alternatively altering the balance between lytic and non-lytic mechanisms of action. Despite their potentially important physiological and pharmacological implications, these mechanistic scenarios remain largely unexplored and require further experimental validation and computational modelling.

### 3.4. Beyond Pore Formation: Non-Lytic and Membrane Remodeling Activities of AMPs

Membrane-active peptides eventually do not cause only immediate membrane disruption or extensive bilayer damage. In many cases, their interaction with lipid membranes results in progressive increases in permeability mediated by transient and highly dynamic membrane defects rather than by the formation of stable pore structures [139,155]. Such short-lived permeabilization events are thought to originate from local disturbances in lipid organization induced by peptide binding and insertion. These perturbations can promote the formation of packing defects and transient phase heterogeneities within the bilayer, reflecting a complex interplay among peptide accumulation, lipid rearrangement, and membrane mechanical properties.

A well-studied example of this mechanism is provided by magainin 2, whose activity has been linked to the generation of asymmetric membrane stress. Insertion of amphipathic peptides into the outer membrane leaflet increases its effective surface area, creating an imbalance between the two leaflets of the bilayer. The resulting mechanical tension may destabilize membrane architecture, ultimately promoting stochastic formation of transient pores and local membrane rupture. Importantly, both the dimensions and persistence of these defects are strongly dependent on peptide concentration, emphasizing the cooperative nature of peptide-induced membrane perturbation [145].

Beyond the generation of membrane tension, certain peptides can modify bilayer thickness and induce curvature stress, thereby lowering the energetic cost of transient defect formation [143]. Such membrane openings generally exhibit limited stability and tend to disappear following lipid reorganization processes, including transbilayer lipid redistribution and relaxation of local membrane stress.

A growing body of evidence from differential scanning calorimetry, NMR spectroscopy, cryo-electron microscopy, and related biophysical approaches indicates that interactions between cationic AMPs and anionic membrane lipids can drive local peptide accumulation and membrane crowding, leading to substantial reorganization of membrane structure and dynamics [120,123,156]. Under these conditions, membrane organization can be substantially altered, promoting lateral phase separation and the emergence of lipid domains that themselves contribute to membrane destabilization [157]. Consistent with this concept, atomic force microscopy studies have demonstrated that small cationic peptides may induce the formation of cardiolipin-enriched membrane domains accompanied by reduced acyl-chain ordering and altered bilayer organization [142,144]. Such lipid redistribution processes may have consequences extending beyond membrane integrity, including displacement of peripheral membrane proteins and disruption of cellular functions associated with energy metabolism and cell envelope biosynthesis [120].

Importantly, these processes are closely linked to classical AMP mechanisms. Local enrichment of anionic lipids, membrane crowding, and domain formation may lower the energetic barrier for pore formation and other membrane-disruptive events, underscoring the interconnected nature of AMP-induced membrane responses [146]. From the perspective of the present review, such membrane-sensitive processes may be particularly susceptible to modulation by APAP-induced changes in lipid organization, membrane fluidity, and bilayer mechanics, potentially amplifying or redirecting AMP activity through indirect physicochemical mechanisms.

### 3.5. Coupling Between Translocation and Permeabilization

It is increasingly recognized that peptide translocation across membranes and membrane permeabilization are not mutually exclusive processes but may share common mechanistic features. Both processes often exhibit cooperative behavior, requiring a threshold concentration of peptides at the membrane surface. Recent studies have demonstrated that certain peptides are capable of both translocating across membranes and forming transient pores, with these processes occurring on different timescales [158,159]. For example, the peptide PuroA has been shown to simultaneously penetrate the membrane and induce pore formation, with a temporal separation between these events [160].

At the molecular level, the outcome of peptide-membrane interaction depends on the balance between kinetic processes—such as peptide binding, insertion, and aggregation—and the time required to accumulate a sufficient number of peptide molecules at the membrane interface. This highlights the importance of studying the kinetics of peptide-membrane interactions. Indeed, time-dependent effects, such as preincubation of membranes with peptides, can significantly influence the observed membranotropic activity. Such kinetic aspects were addressed in studies of the cyclic cationic oligopeptide gramicidin S, where membrane effects varied depending on incubation time [111]. Interestingly, delivery of gramicidin S in lipid vesicles, where interactions with liposomal lipids were of independent interest, was shown to alter the targeting and membranotropic properties of this AMP [82].

A possible cooperative mechanism may involve subtle membrane toxicity and membrane pre-damage induced by APAP. In this context, APAP-mediated non-lytic effects may not immediately disrupt membranes but instead promote membrane pre-conditioning by altering lipid and protein packing, inducing local defects, and modifying the lateral organization of the lipid bilayer. Such effects may arise from documented APAP-associated oxidative stress, lipid peroxidation [3,37,43,73,161], and membrane softening. APAP may additionally alter membrane lipid order parameters and thereby influence membrane phase separation and lateral bilayer organization, potentially further modulating AMP–membrane interactions. In membrane systems approaching a critical destabilization threshold, even relatively small perturbations induced by APAP could produce amplified effects, potentially lowering the energetic barrier for the formation of transient membrane pores.

### 3.6. Membrane-Dependent Synergistic and Cooperative Effects of AMPs

Cooperative interactions between AMPs constitute an important aspect of their biological activity and have attracted considerable attention in recent years. Increasing evidence indicates that the biological effects produced by combinations of AMPs often exceed the simple sum of the activities of individual peptides, resulting in enhanced antimicrobial efficacy and membrane-disruptive capacity [162,163,164]. More broadly, the principles underlying peptide–peptide cooperation may also be relevant for interactions between AMPs and other membrane-active compounds, including pharmacological agents capable of modifying membrane physicochemical properties.

Several mechanisms have been proposed to explain AMP synergy. Cooperative accumulation at the membrane surface can increase local peptide concentration and promote formation of membrane-active assemblies [165,166]. Membrane-bound peptides may also facilitate insertion of additional molecules, while hetero-oligomer formation and collective modulation of membrane properties can enhance membrane disruption [137,167]. A classical example is the magainin 2–PGLa system, in which heterodimerization promotes membrane insertion and pore formation more effectively than either peptide alone [166].

A recent quantitative model suggests that synergistic activity may arise from cooperative membrane association, whereby binding of one peptide facilitates recruitment of another and accelerates assembly formation at the membrane surface [164]. This framework highlights membrane properties as key determinants of synergistic behavior. Consequently, APAP-induced membrane remodeling could alter cooperative binding equilibria, peptide association kinetics, and the concentration thresholds required for AMP synergy.

An important characteristic of many synergistic AMP systems is the existence of cooperative threshold behavior. At subcritical surface concentrations, peptides may adsorb onto membranes with only limited effects on membrane integrity. However, once a critical peptide density is reached, membrane responses often become highly nonlinear, resulting in abrupt increases in permeabilization and membrane destabilization [137,166]. Such threshold phenomena are commonly associated with collective peptide insertion, progressive membrane thinning, accumulation of elastic stress, and the emergence of transient or stable membrane defects. Consequently, membrane disruption should be viewed not merely as a function of peptide concentration but as an emergent property of the peptide–membrane system.

The manifestation of synergistic effects is strongly influenced by membrane composition and structural heterogeneity. Lateral lipid organization, phase separation, and the presence of membrane microdomains may promote local peptide enrichment and alter the stability of peptide-induced membrane structures [81,111,120,144]. Such observations suggest that the membrane itself acts as an active participant in synergistic interactions rather than a passive target. Within this framework, APAP-induced alterations in membrane organization, lipid packing, fluidity, or domain architecture may potentially shift cooperative thresholds and modify the efficiency of peptide self-assembly, insertion, and pore formation. Although direct experimental evidence for such effects is currently lacking, membrane-mediated modulation represents a plausible mechanistic basis for potential AMP–APAP synergy and warrants systematic investigation.

## 4. Future Perspectives: Experimental Approaches to Address Current Knowledge Gaps

The lack of experimental evidence regarding the combined effects of acetaminophen and AMPs represents a significant knowledge gap. To address this issue, a series of prioritized experimental approaches should be considered.

First, in vitro studies using mammalian cell lines should evaluate the cytotoxicity of acetaminophen and selected AMPs administered individually and in combination. Assessments of cell viability, membrane integrity, oxidative stress, lipid peroxidation, protein modifications, and apoptosis would help determine the extent of additive or synergistic cellular damage induced by combined exposure.

**Figure 3:**
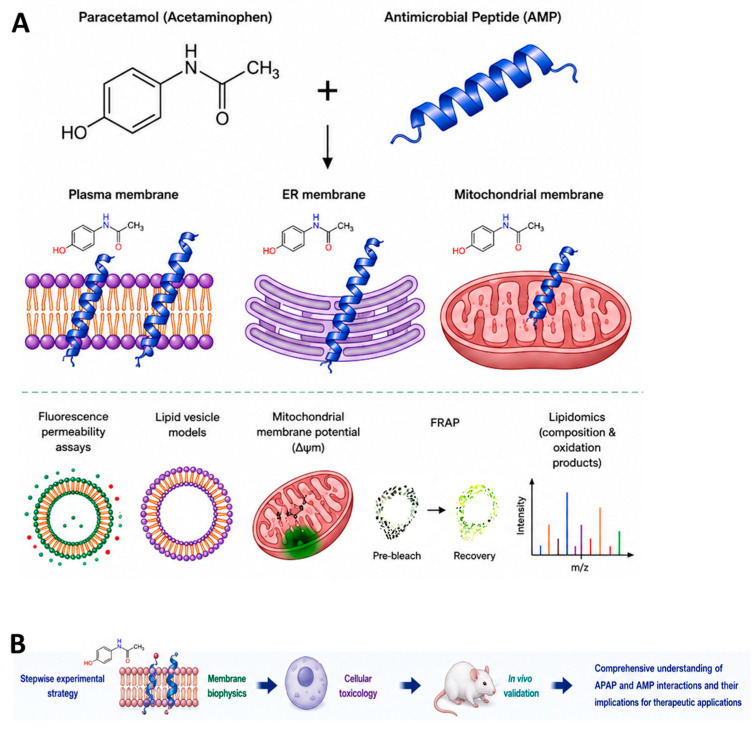
Membrane-specific and general experimental approaches for investigating potential interactions between acetaminphen (APAP) and AMPs. (**A**) Membrane-focused studies should evaluate the effects of APAP and AMPs on the plasma membrane, endoplasmic reticulum (ER) membrane, and mitochondrial membrane to determine whether combined exposure alters membrane integrity, permeability, and biophysical properties. Representative experimental approaches include fluorescence-based membrane permeability assays, lipid vesicle models, measurements of mitochondrial membrane potential (ΔΨm), fluorescence recovery after photobleaching (FRAP), and lipidomic profiling of membrane composition and lipid oxidation products. (**B**) Stepwise experimental framework proposed to address current knowledge gaps from the molecular and cellular levels to whole-organism outcomes. The figure was generated with the assistance of an AI-based image generation tool (GPT-5.3). The authors validated all scientific content and take full responsibility for the accuracy and integrity of the figure.

Second, membrane-specific investigations should assess alterations in plasma, endoplasmic reticulum, and mitochondrial membranes. Fluorescence-based membrane permeability assays, lipid vesicle models, measurements of mitochondrial membrane potential, fluorescence recovery after photobleaching (FRAP), and lipidomic profiling of membrane composition and oxidation products could be complemented by confocal microscopy using membrane- and organelle-specific probes, electron microscopy analyses, and atomic force microscopy. Together, these approaches could reveal whether acetaminophen modifies AMP–membrane interactions, alters membrane fluidity and mechanical properties, or enhances membrane destabilization and organelle damage. A schematic representation of the proposed experimental framework is presented in Figure 3A.

Third, mechanistic studies, including appropriate animal models, should focus on oxidative stress, lipid peroxidation, mitochondrial dysfunction, and inflammatory signaling pathways, which are central to acetaminophen toxicity and may be influenced by AMP exposure. The quantification of reactive oxygen species, ATP production, mitochondrial membrane potential, respiratory chain activity, calcium homeostasis, mitochondrial dynamics (fusion/fission), and markers of mitochondrial damage would provide valuable insights into the shared mechanisms underlying toxicity induced by combined exposure.

Finally, in vivo studies using appropriate animal models should be conducted to evaluate the physiological relevance of combined exposure under clinically relevant conditions and using therapeutically relevant as well as overdose-associated concentrations of acetaminophen. Such studies should consider different routes of administration, exposure durations, pharmacokinetic profiles, and clinically relevant AMP dosages. Particular attention should be given to conditions associated with increased susceptibility to toxicity, such as pre-existing liver dysfunction, inflammatory states, infectious diseases requiring AMP-based therapies, advanced age, and polypharmacy. These investigations would help determine whether interactions observed in vitro translate into systemic toxicological outcomes and clinically significant adverse effects.

A stepwise experimental strategy integrating membrane biophysics, cellular toxicology, and in vivo validation would provide a comprehensive framework for understanding the potential interactions between acetaminophen and AMPs and their implications for therapeutic applications (Figure 3B).

## 5. Clinical Relevance and Potential Translational Implications of APAP–AMP Interactions

The mechanisms discussed in this review could have important implications for clinical practice, particularly in patient populations simultaneously exposed to APAP and AMPs or other membrane-active antimicrobial agents. Although direct clinical evidence for APAP–AMP interactions is currently lacking, the convergence of their biological effects at the level of cellular membranes raises several safety considerations.

Although APAP is generally considered safe at therapeutic doses, overdose, chronic supratherapeutic exposure, malnutrition, advanced age, alcohol abuse, and pre-existing liver disease substantially increase the risk of toxicity. Under these conditions, APAP-induced mitochondrial dysfunction, oxidative stress, membrane destabilization, and depletion of antioxidant defences may create a cellular environment that is more susceptible to additional membrane-targeting insults, including the therapeutic and off-target effects of AMPs.

Several AMP-based therapeutics and peptide-derived antimicrobial formulations are currently undergoing clinical development [49,108,112]. Although the literature often refers to “AMPs in clinical use,” this term frequently encompasses naturally derived antimicrobial antibiotics with membrane-targeting mechanisms (e.g., polymyxins and gramicidins) rather than classical host-defense peptides such as LL-37 or defensins. Strictly speaking, only a limited number of host-defense peptide-based AMPs have progressed to systemic clinical use or advanced clinical evaluation [168]. Table 4 lists the most used AMPs in the clinic.

In addition to the clinically approved peptide-based antimicrobials listed in Table 3, several other antimicrobial peptides, including pexiganan, omiganan, brilacidin, and LL-37 derivatives, have advanced to Phase II or III clinical trials but have not yet received regulatory approval [168,169].

There is no common therapeutic AMP concentration, at the same time for the majority of AMPs MIC is usually around 1–20 μM; membrane effects on eukaryotic cells often occur at 10–100 μM, depending on the peptide. Hemolytic concentrations for some AMPs can be much higher than MIC, but for some membranotropic peptides they overlap with therapeutic [170,171,172]. That is why even a small shift in the threshold of membrane activity caused by APAP could theoretically be important. And this supports our hypothesis very well.

While many AMPs exhibit favourable selectivity toward microbial membranes, off-target interactions with host–cell membranes have also been reported, particularly at elevated concentrations or under pathological conditions associated with membrane remodeling and oxidative stress [49,112]. Consequently, patients receiving AMP-based therapies may represent a population in which APAP-induced membrane alterations could modify peptide activity, biodistribution, toxicity, or cellular uptake.

Particular attention should be given to critically ill patients and individuals with severe infections, sepsis, chronic inflammatory disorders, acute liver injury, or malnutrition. These conditions are frequently associated with oxidative stress, altered membrane composition, mitochondrial dysfunction, and increased permeability of biological barriers [5,92]. Because such alterations may influence both APAP toxicity and AMP–membrane interactions, the possibility of additive or synergistic adverse effects must be considered. Monitoring of liver function, oxidative stress biomarkers, and indicators of mitochondrial injury may therefore become relevant in future clinical studies evaluating AMP-containing therapeutic regimens.

The findings reviewed here are also relevant for drug development. The membrane-modifying properties of APAP observed under toxic conditions suggest that membrane composition, lipid oxidation status, and mitochondrial integrity should be considered during the preclinical evaluation of novel AMPs, membrane-active therapeutics, and AMP-based delivery systems [72,81,173,174].

Finally, the identification of membrane-associated mechanisms common to both APAP toxicity and AMP activity supports the development of precision-medicine approaches aimed at identifying patient populations at increased risk of adverse outcomes. Future translational studies integrating toxicology, membrane biophysics, lipidomics, and clinical pharmacology will be essential to determine whether the mechanistic interactions proposed in this review have measurable consequences in clinical practice.

## 6. Discussion

The development of new diagnostic and therapeutic strategies depends on, and may be further advanced by, detailed knowledge of the molecular mechanisms underlying APAP toxicity under both normal and altered nutritional conditions. In this context, potential drug–drug interactions may represent an additional important modulatory factor. Among the very limited literature evidence regarding interactions between APAP and AMPs, direct binding between acetaminophen and a cyclic 7-mer peptide has been investigated. Functional interactions of the peptide with APAP in solution were demonstrated, and molecular dynamics simulations were performed to characterize these interactions [175]. In another study, the AMP cathelicidin was shown to promote liver repair after acetaminophen-induced liver injury in a mouse model [176]. Although the exact molecular mechanisms remain unknown, these early observations suggest potentially cooperative effects and support the need for broader investigation of APAP–AMP interactions.

A range of drug delivery strategies, including nanoliposomal formulations, may offer approaches to reduce APAP-associated toxicity [173]; therefore, comprehensive knowledge of APAP–lipid membrane interactions is essential for their rational design. Similar considerations are directly applicable to studies of AMPs, particularly regarding their membrane interactions and delivery systems.

Summarizing the controversies and conflicting evidence in APAP toxicity research, the main unresolved issues concern the relative contribution of the numerous mechanisms leading to hepatocellular injury. While the formation of the reactive metabolite NAPQI and mitochondrial dysfunction are generally accepted as central events, the extent and impact of downstream oxidative processes continue to be debated. One of the most contentious issues concerns the role of LPO. Although LPO has been extensively documented during APAP-induced liver injury, many studies suggest that it contributes relatively little to hepatotoxicity compared with peroxynitrite-mediated protein modification and mitochondrial dysfunction, which are considered the dominant mechanisms. In contrast, other investigations have demonstrated protective effects of interventions targeting lipid-derived aldehydes or enhancing aldehyde detoxification systems, suggesting that localized mitochondrial LPO and reactive aldehydes, such as 4-HNE, may substantially amplify cellular damage. These apparently conflicting observations may reflect differences in experimental models, the timing of sample collection, species-specific responses, or the distinction between global and compartmentalized oxidative damage.

Another important pathophysiological issue concerns the role of inflammation. Although sterile inflammation is recognized as an important consequence of APAP overdose, immune activation has been reported to exert both detrimental and beneficial effects. Early recruitment of neutrophils, macrophages, and other innate immune cells may enhance tissue injury through cytokine production and oxidative stress, whereas the same cell populations are also essential for debris clearance, tissue remodeling, and liver regeneration. Consequently, inflammation should not be viewed exclusively as a pathogenic process but rather as a dynamic response whose overall impact depends on disease stage and cellular context.

Considerable uncertainty also exists regarding the translation of findings obtained in experimental animal models to human disease. Most mechanistic knowledge derives from murine studies, whereas significant differences in hepatic metabolism, immune regulation, antioxidant capacity, and mitochondrial responses have been documented between rodents and humans. These interspecies differences may explain why some therapeutic strategies that demonstrate efficacy in experimental models have shown limited clinical success.

The greatest knowledge gap addressed in the present review concerns the potential interaction between APAP and AMPs. Although both agents can influence membrane organization, mitochondrial function, oxidative stress, and inflammatory signaling, direct experimental evidence regarding their combined effects is currently lacking. Whether co-exposure results in additive toxicity, synergistic membrane destabilization, or, conversely, protective interactions remains entirely unknown.

Addressing these controversies will require standardized experimental protocols, greater use of human-relevant cellular and organoid models, advanced lipidomic and proteomic approaches, and systematic studies evaluating APAP–AMP co-exposure (see Section 4. Future Perspectives: Experimental Approaches to Address Current Knowledge Gaps). Such investigations will be essential for distinguishing universally conserved mechanisms from context-dependent phenomena and for improving the translational relevance of future studies.

In summary, current evidence indicates that APAP toxicity arises from a multifactorial disruption of cellular homeostasis rather than from a single direct mechanism. Central to this process is membrane damage at multiple cellular levels, including mitochondrial membranes, where membrane potential collapse and lipid peroxidation initiate cell death pathways, and the plasma membrane, where ion pump dysfunction promotes osmotic imbalance and eventual cell lysis. In this context, the presence of AMPs targeting similar cellular compartments may produce unforeseen biological consequences (Figure 2). Moreover, the synergistic interaction between APAP exposure and protein deficiency may create an “ideal storm” characterized by glutathione depletion, activation of toxic metabolic pathways, and impaired cellular regenerative capacity. These findings highlight the need for increased clinical caution when prescribing paracetamol to malnourished patients and underscore the importance of further studies addressing the potential combined toxicity of APAP and AMPs.

## 7. Conclusions

Acetaminophen toxicity involves complex membrane-associated mechanisms, including mitochondrial dysfunction, oxidative stress, and lipid peroxidation. AMPs, while therapeutically promising, may also induce off-target effects on host cell membranes. Combined exposure to APAP and AMPs may therefore produce additive or synergistic toxic effects, although such interactions remain largely unexplored. Further studies are needed to clarify their biological and clinical relevance.

## Figures and Tables

**Table 1:** Major acetaminophen metabolic pathways and their relative contributions under therapeutic and overdose conditions. Note, the fraction of APAP metabolized via a given pathway vary across studies.

Metabolic Pathway	Enzyme Systems	End Product	Percentage of APAP Metabolized via Each Pathway (Therapeutic Conditions)	Effect of APAP Overdose	Reference
Glucuronidation	UGT1A1, 1A6, 1A9	APAP-Glucuronide	50–60	Pathway Saturation	[15]
Sulfation	SULT1A1, 1A3	APAP-Sulfate	25–35	Critical Substrate Exhaustion	[15]
Oxidation (CYP enzymes)	CYP2E1, 1A2, 3A4	NAPQI (Reactive)	5–10	Massive Flux Increase	[29,31]
GSH Conjugation	GST (Alpha, Mu, Pi)	NAPQI-Glutathione	5–10	GSH Pool Depletion	[15,32]
Protein Covalent Binding	Direct NAPQI binding	Protein adducts with NAPQI	negligible	Massive Adduct Formation	[16,34]

**Table 2:** Impact of APAP/NAPQI on Membrane Parameters.

Membrane Indicator	APAP/NAPQI Impact	Consequence for Cells	Reference
Na^+^/K^+^-ATPase Activity	Reduction by >50%	Osmotic Swelling, Potential Loss	[69]
Ca^2+^ Permeability	Increase (via TRPM2)	Calpain Activation, Cytoskeletal Decay	[71]
Pure phospholipid membranes	Alteration of biochemical and biophysical properties	unknown	[72]
Lipid Fluidity	Increase	Disrupted Signaling and Enzyme Activity	[83]
Bending Rigidity	50% Reduction	Vulnerability to Rupture and Lysis	[83]

**Table 3:** Factors Influencing APAP Toxicity during Protein Malnutrition.

Factor	Status with Adequate Nutrition	Status with Protein Deficiency	Risks	Reference
GSH Level	High (Stable)	Reduced	Instant Loss of Protection	[89]
CYP2E1 Activity	Baseline	Reduced	unknown	[85]
Sulfation Rate	Optimal	Retarded (Sulfate lack)	APAP Accumulation	[90]
Liver Regeneration	Effective	Inhibited (Material lack)	Progression to Liver Failure	[91]

**Table 4:** Clinically used antimicrobial peptides and peptide-derived antimicrobial agents.

Peptide	Status	Type
Daptomycin	FDA/EMA approved	Systemic
Colistin	approved	systemic/inhalable
Polymyxin B	approved	systemic/topical
Gramicidins (including Gramicidin S in several post-soviet countries)	topical	Topical
Nisin	Approved by FDA for veterinary and as a food preservative	Topical

## Data Availability

No new data were created or analyzed in this study.

## Abbreviations

The following abbreviations are used in this manuscript:

APAP	Acetaminophen (paracetamol)
AIF	apoptosis-inducing factor
AMPs	Antimicrobial peptides
AM404	N-arachidonoylphenolamine
ADP	adenosine diphosphate
ATP	adenosine triphosphate
AMP	adenosine monophosphate
ALDH2	aldehyde dehydrogenase 2
ALT	alanine aminotransferase
cAMP	cyclic AMP
COX	cyclooxygenase
CYP	cytochrome P450
cryo-TEM	cryogenic transmission electron microscopy
DAMPs	damage-associated molecular patterns
DILI	drug-induced liver injury
Drp1	dynamin-related protein 1
DOPC	1,2-di-(octadecenoyl)-sn-glycero-3-phosphocholine
DLS	dynamic light scattering
ETC	electron transport chain
4-HNE	4-hydroxynonenal
GSH	reduced glutathione
GSSG	oxidized glutathione
GST	glutathione-S-transferases
GPx	glutathione peroxidase
15-HETE	15-hydroxyeicosatetraenoic acid
JNK	c-Jun N-terminal kinase
LUVs	large unilamellar vesicles
LPO	lipid peroxidation
MIC	Minimal inhibitory concentration
MRP2	multidrug resistance–associated protein 2
MCJ	methylation-controlled J protein
MCFU	mitochondrial electrogenic Ca^2+^/Fe^2+^ uniporter
MRPs	multidrug resistance-associated proteins
mtDNA	mitochondrial DNA
mPTP	mitochondrial permeability transition pore
NAC	N-acetylcysteine
NLS	nuclear localization signals
NSE	neutron spin-echo
NAPQI	N-acetyl-p-benzoquinone imine
nNOS	neuronal nitric oxide synthase
PPARγ	Peroxisome Proliferator-Activated Receptor Gamma
ROS	reactive oxygen species
SOD	superoxide dismutase
SANS	small-angle neutron scattering
SAXS	small-angle X-ray scattering
TRPV1	transient receptor potential vanilloid 1
TRPM2	Transient Receptor Potential Melastatin 2
UGT	UDP-glucuronosyltransferase
VDAC	voltage-dependent anion channel
